# Where do people look when walking up and down familiar staircases?

**DOI:** 10.1167/jov.23.1.7

**Published:** 2023-01-12

**Authors:** Andrea Ghiani, Liz R. Van Hout, Joost G. Driessen, Eli Brenner

**Affiliations:** 1 Department of Movement Sciences, Vrije Universiteit Amsterdam, The Netherlands

**Keywords:** gaze, stairs, daily life

## Abstract

Many activities in daily life do not impose strict requirements on gaze. We investigated gaze when walking up and down staircases within one's own house. We anticipated that using a variety of staircases in different environments and not informing participants that stair climbing was the focus of investigation might provide a description of gaze behavior that is closer to that used in our daily life than doing so under circumstances in which the focus is explicitly and exclusively directed at the stairs. We analyzed several measures, including the order in which participants fixated the steps. We confirmed that people often look at the steps sequentially, but found that they often made fixations back to steps they had already fixated. They also regularly skipped looking at several steps to fixate further ahead. On average, they directed their gaze at about half the steps. They looked further ahead when ascending than when descending staircases. Overall, the results are similar to those found under highly constrained laboratory conditions, although we do report some differences. One such difference is a tendency to fixate fewer steps. Another is that participants fixated steps that were less far ahead when descending staircases. We also introduced some new analyses that may help understand gaze behavior during stair climbing.

## Introduction

Many studies have shown that gaze is directed at items that are critical for the task at hand (Hayhoe, Shrivastava, Mruczek, & Pelz, 2003; Land & Hayhoe, 2001; Sullivan, Ludwig, Damen, Mayol-Cuevas, & Gilchrist, 2021). However, in daily life, there are many situations in which it is not evident where gaze should be directed because the task does not require constant visual guidance and there may be reasons to look elsewhere. For instance, when walking it might not always be necessary to look at the ground in front of you. When foot placement is critical, such as when walking on rough terrain, people need to look at the ground in front of them to find suitable places to place their foot (Marigold & Patla, 2007; Matthis, Yates, & Hayhoe, 2018; Patla & Vickers, 2003; ’t Hart & Einhäuser, 2012). However, when walking on flat surfaces in more familiar environments, such as one's own house, people may not need to constantly direct their gaze at the path just in front of them. This does not mean that they no longer use vision to guide their movements, but in such situations peripheral vision might be precise enough and it may be more useful or entertaining to look elsewhere.

Several papers on stair walking report that people spend most of their time looking at steps ahead of them on the walking trajectory, presumably to extract features that are crucial for correct foot placement (Miyasike-DaSilva, Allard, & McIlroy, 2011; Zietz & Hollands, 2010). However, other papers report that central vision is not essential for safe foot landings. Den Otter, Hoogwerf, and van der Woude (2011) reported that some steps that are stepped on are never fixated, suggesting that the gaze-stepping coupling that is found in some constrained ground walking studies (Matthis et al., 2018; Patla & Vickers, 2003) is not observed during stair walking (Den Otter et al., 2011). In general, people can guide their foot placement using peripheral vision when they must direct their gaze elsewhere, such as towards a mobile phone (Ioannidou, Hermens, & Hodgson, 2017) or to perform a secondary task (Miyasike-daSilva & McIlroy, 2012; Miyasike-daSilva & McIlroy, 2016).

Fixating steps is not even a prerequisite for a safe transition between the floor and the first step (Miyasike-daSilva & McIlroy, 2012; Miyasike-daSilva & McIlroy, 2016). The duration of fixations and number of fixations on the first step are similar to those on other steps (Miyasike-DaSilva et al., 2011). However, there are reports that performance on a second, cognitively demanding task is poorer when transitioning from the floor to the first steps than when walking up or down the central part of the staircase (Miyasike-daSilva & McIlroy, 2012; Telonio, Blanchet, Maganaris, Baltzopoulos, Villeneuve, & McFadyen, 2014). Moreover, when visibility near the foot is occluded, foot clearance is greater for the first two steps than for later steps (Graci, Rabuffetti, Frigo, & Ferrarin, 2017). Foot clearance is the foot's height during the swing phase, which is often considered as a measure of the adopted margin of safety against tripping. Thus, although it may not be necessary to fixate the first steps, there is reason to believe that visual guidance is particularly important for this part of the staircase.

So far, stair climbing has mainly been studied in highly controlled experimental settings with clear instructions confirming that stair climbing is the primary task. However, stairs are usually encountered within more complex settings without the staircase having particular relevance other than being part of the path. There is evidence that experimental settings and task requirements may influence gaze (Ballard & Hayhoe, 2009; Castelhano, Mack, & Henderson, 2009; Dicks, Button, & Davids, 2010; Foulsham, Walker, & Kingstone, 2011; Zeuwts, Vansteenkiste, Deconinck, van Maarseveen, Savelsbergh, Cardon, & Lenoir, 2016). The goal of the current study was to provide a description of gaze behavior during stair climbing under circumstances that are as close as possible to those in our daily life. We examined gaze on a variety of staircases in various familiar but uncontrolled environments by asking participants to perform a navigation task that we knew would require them to use stairs within their own house. Participants were unaware that stair climbing was the focus of investigation. We examined whether steps were fixated sequentially, as has often been reported (Miyasike-DaSilva et al., 2011; Zietz & Hollands, 2010), and how many steps ahead people usually looked when ascending and descending the staircase. We also examined whether some steps were fixated more frequently than others under such circumstances, despite this not being so in a highly controlled unfamiliar environment (Miyasike-DaSilva et al., 2011). Finally, as the transition between the floor and the staircase is probably where one needs to rely most on visual guidance (Graci et al., 2017; Miyasike-daSilva & McIlroy, 2012; Telonio et al., 2014), we examined where gaze was directed just before reaching the staircase.

## Methods

### Participants

Thirty participants (17 women; age range = 18–60 years; mean age = 30 years) with normal or corrected-to-normal vision and no difficulties walking took part in the experiment. The experiment was conducted in accordance with the approval by The Scientific and Ethical Review Board of the Faculty of Behaviour and Movement Sciences (file VCWE-2021-035), which included all participants providing written informed consent.

### Setup and data collection

Participants were engaged in a navigation task in their own house, without knowing that stair climbing was the focus of investigation. They were asked to walk around their house. To increase the probability of taking the stairs, the experimenters suggested to the participants that they might like to include visiting certain rooms in the house that required taking a staircase, but without mentioning the staircase. For some participants, there was no staircase in the actual apartment, but there was a staircase in the building that included their apartment. In that case, the experimenters suggested including walking out of the apartment. We did not explicitly check whether participants were aware that we were interested in staircases, but at the end we described our goals and participants never mentioned that they suspected that the staircases were important. The uncontrolled setting means that we could not forbid participants to skip steps. Because it is not evident how to interpret gaze when steps are skipped, we identified and excluded any cases in which participants skipped steps. Participants were obviously very familiar with the environment (having lived in that environment for a median of 11.5 months). The experimenter did not follow or observe them as they walked around. The staircases had different numbers of steps (median = 13; range = 4–19). Gaze behavior was measured with the Pupil Invisible glasses (Pupil Labs, GmbH) at a sampling rate of 30 Hz. The Pupil Invisible glasses are a calibration-free wearable eye tracker that can provide a good estimate of gaze position under realistic and highly variable conditions. The scene camera field of view is 82 degrees × 82 degrees (a description of the Pupil Invisible glasses’ performance can be found here: arxiv:2009.00508).

Once the recording started, participants put the recording phone connected with the glasses in their pocket and started walking freely. Participants encountered either one or two staircases during their journey, each of which was encountered twice: once when going up and once when going down. We considered all encountered staircases in our analysis, leading to a total of 44 staircases both when ascending and when descending.

### The steps

We conducted a frame-by-frame analysis (manual annotation by the first author based on the scene images with superimposed gaze locations) to determine which steps were fixated. This gave rise to several ambiguous situations that will be discussed in a later section. Scene videos and gaze positions were visualized with a custom-built Python script. Much of the manual frame-by-frame analysis could have been done using the visualization software provided by Pupil Labs (Pupil Player, Pupil Labs, GmbH), but some analyses (distance judgments and step estimates) were easier to perform with our script. The gaze data were not filtered before being visualized.

After localizing sections of the scene videos that contained a staircase, gaze during those periods was subjected to a frame-by-frame analysis to label each fixated structure. Different staircases were visible from different moments and from different perspectives for different participants. The manual frame-by-frame analysis started at the first fixation on any step and ended after the last fixated step (mean ± standard deviation of the duration was 5.4 ± 2.1 seconds when ascending and 5.9 ± 2.5 seconds when descending). A step was considered to have been fixated if gaze was directed at about the same part of the step for at least two frames (about 66 ms). Start and end times of blinks were detected by inspecting video images of the eyes (the images that are used to estimate gaze). Blinks were discarded from the frame-by-frame analysis (the mean ± standard deviation of the time discarded due to blinks was 0.17 ± 0.07 seconds when ascending and 0.22 ± 0.08 seconds when descending).

**Figure 1.**
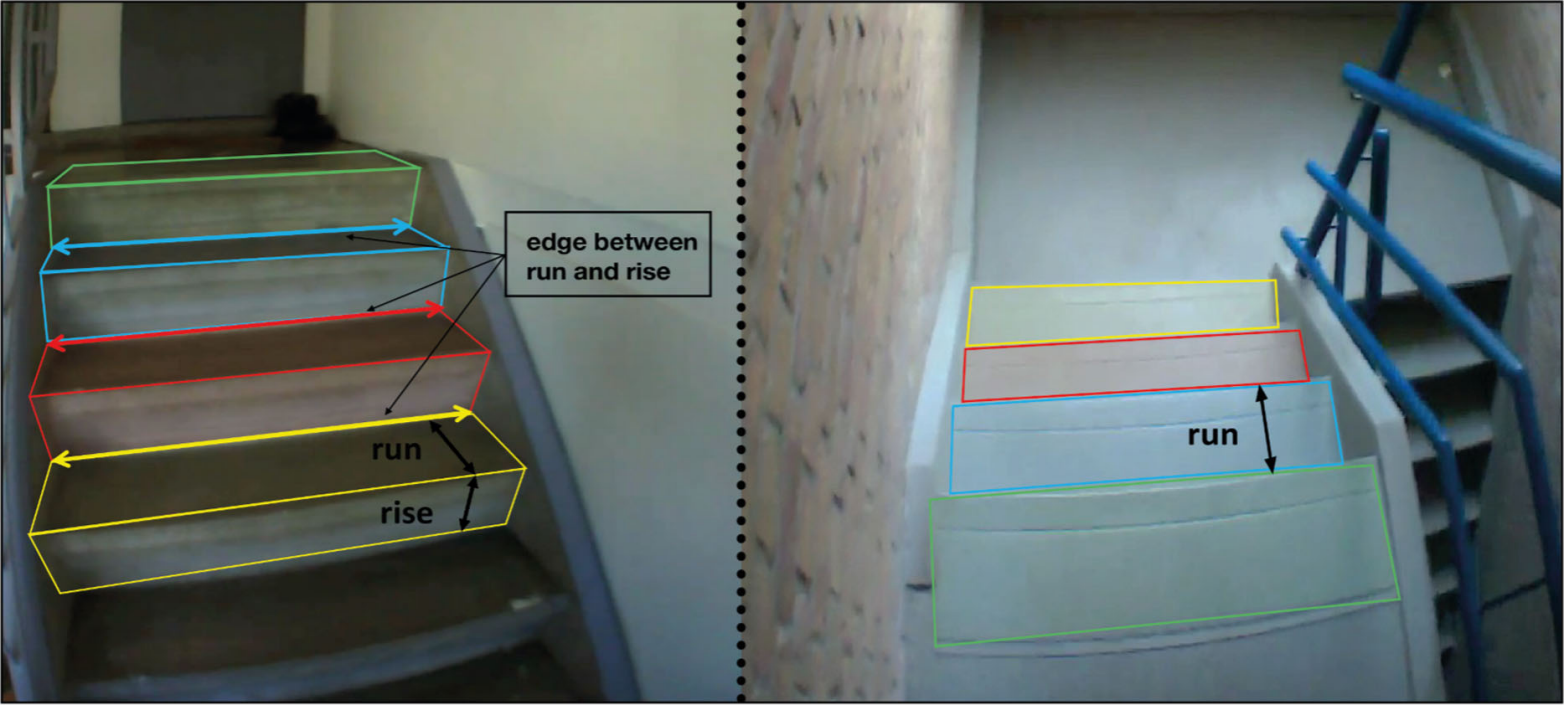
A step is defined by its run (horizontal surface) and rise (vertical surface). When gaze was on the edge between the run of one step and the rise of the next it was assigned to the former. When descending, the rise is not visible.

Each fixation was either classified by a step number or as “elsewhere” if gaze was not directed at a step. If sequential fixations were on the same step, separated by a saccade along that step, the fixations were merged under the same step number. Likewise, if multiple fixations were made on non-steps, those fixations were merged under the same label “elsewhere.” We did not differentiate between structures at which gaze was directed when looking elsewhere, so looking at the handrail was also classified as “elsewhere.” For each staircase, this procedure led to a sequence of fixated steps interleaved by some periods of fixating elsewhere. Figure 1 indicates how each step was defined.

### Data analysis

#### Gaze sequence, fraction of steps that were fixated and steps looked ahead

In order to provide a general description of the gaze sequence during stair climbing, we examined how gaze transitioned between steps across successive fixations by computing the number of steps between pairs of successively fixated steps. The sign of this difference indicates the direction of the next fixation: shifting gaze to a step that one will reach later is positive and shifting gaze to a step that one will reach earlier is negative. Transitions were treated separately if gaze shifted elsewhere before shifting back to one of the steps. In that case, there could also be no steps between pairs of successively fixated steps: gaze could shift away from a step and then back to the same step. For this analysis, it was important to have reliable gaze data during the whole period on the staircase and it was essential to have at least two fixations on two different steps. No steps were fixated when descending three staircases, and only one step was fixated when ascending two and descending one staircase. The illumination was too poor to detect the steps with confidence when ascending one staircase and descending four staircases. The participant's hair was in front of the scene camera when descending one staircase. It was evident from the scene video that a participant was skipping steps when ascending two staircases. Thus, in total, five staircases when ascending and nine staircases when descending were excluded from the analysis of gaze sequences, bringing the total number of ascended staircases to 39 and of descended staircases to 35. This led to five participants being excluded when descending and one participant when ascending.

The six cases in which steps could not be coded (due to bad illumination or because of hair in front of the scene camera) and the two cases in which the participant skipped one or more steps were also excluded from the analysis of the fraction of steps that were fixated. The staircases for which one step or no steps were fixated were included in this analysis, leading to a total of 41 staircases when ascending and 39 staircases when descending. The fraction was obtained by dividing the number of fixated steps in each staircase by the total number of steps in that staircase. Additionally, we determined the fraction of fixations in which gaze was directed at each of the first four and last four steps by simply counting how often each of these steps was fixated and dividing this by the number of staircases. We decided to consider the first four and last four steps as these were defined for all the staircases (as already mentioned, there was one staircase with only 4 steps).

Finally, we determined how many steps participants looked ahead while on the staircase. To do this, we had to estimate which step participants were stepping onto. This was judged from the output of the inertial measurement unit (*IMU*) in the eye tracker. The *IMU* provided information about rotations around three orthogonal axes and accelerations in three orthogonal directions. Assuming that gravity is always directed downward and has a constant value of 9.81 m/s^2^, we used this information to estimate the head's vertical displacement and pitch angle (for details on how we did so, please see the *readIMU* Python script provided at the location indicated in the Data Availability Statement). We considered the moment at which the head's position was lowest during each stride as the moment that the foot was placed stably. We determined where the participant was looking at that moment, and defined the number of steps looked ahead by subtracting the number of the step onto which participants were stepping (the one on which their foot was placed stably) from the number of the fixated step. If no step was fixated at the moment that the foot was placed stably, the number of steps looked ahead was not computed for that foot placement. Following this procedure, participants looked at zero steps ahead if their gaze was on a step when they placed their foot on it. Note that this is a slightly different procedure from that described in an earlier study (Zietz & Hollands, 2010), mainly due to a different way of defining the step that participants were stepping onto. This leads to a difference of about one step: looking five steps ahead according to our definition will usually correspond to looking four steps ahead according to Zietz & Holland's definition. We only computed this measure for foot placements for which a step was fixated and our estimate of which step the person was standing on could be determined reliably from the scene video. Consequently, only fixations from 27 staircases when going up and 23 staircases when going down contributed to this measure.

#### Gaze when approaching the staircase

Because directing one's gaze at the transition between the floor and the staircase may be particularly important, we also determined where gaze was directed as participants approached the staircase. We considered all moments at which the edge of the first step was visible in the scene image during the two seconds before the foot was placed stably on the first step. Note that the moment the foot was placed stably on the first step is when participants stepped onto the first physical step when ascending a staircase. It is when they stepped onto the last part of the floor when descending a staircase. We determined how far gaze was directed from the edge of the first step, whereby whether gaze was above or below the edge of the first step was considered by assigning a negative value to the distance if gaze was directed below the edge of the first step. Because the edge of the first step had to be visible for this analysis, we obtained data for different time intervals for each staircase. In some cases, we obtained no data at all because the first step was never visible in the scene video. This left us with 20 staircase ascents and 23 staircase descents.

### Ambiguous situations

**Figure 2.**
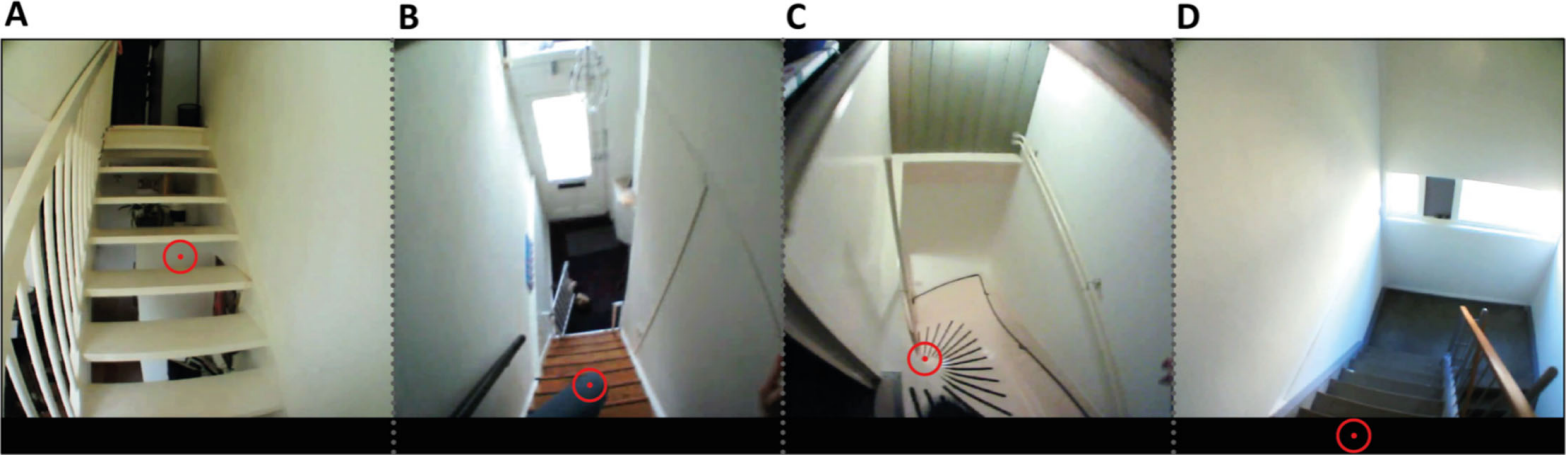
Situations in which coding was somewhat ambiguous when ascending a staircase (**A**), descending a staircase (**B, C**), or both (**D**, only descending is shown). The red dot within the red disk indicates the direction of gaze. This direction was also indicated when gaze was below the scene image (as in **D**), within a black region that was added for this purpose.

The total number of fixated steps was 414 when going up and 302 when going down. We encountered some situations in which the coding of some of these steps was ambiguous. In this section, we explain how we dealt with them and report their frequency of occurrence. One such situation when ascending staircases is that there was sometimes no physical rise (Figure 2A). In the 20 cases in which participants fixated the absent rise, gaze was labeled as if a physical rise were present. When descending staircases, gaze was sometimes temporarily directed at a part of the participant's body that was occluding the steps (Figure 2B). In the 29 cases in which this happened, gaze was classified as being directed at the step behind the body part. An ambiguous situation that arose seven times when looking further ahead while descending curved staircases is that gaze was directed at the point at which the steps converge (Figure 2C). In that case, the resolution of the eye tracker was no longer good enough to be confident about the step that was fixated. Moreover, in that situation, gaze might actually be directed at the inner curve, as occurs when driving or cycling (Land, 1992; Vansteenkiste, van Hamme, Veelaert, Philippaerts, Cardon, & Lenoir, 2014). It is not clear what participants could want to judge about the narrowest part of the step, but, in such situations, we nevertheless determined the fixated step as well as we could. Finally, in some cases, gaze was just below the scene camera's range. Gaze positions appear to be reasonably reliable within a slightly wider range than that of the scene camera, so the fixated step was guessed by mentally extrapolating the image of the staircase within a black stripe that was added to the bottom of the frame to help do so (Figure 2D). Extending the range beyond what was visible in the scene images in this manner allowed us to use all the data for which gaze was outside the scene camera's range. This was the case for 74 steps when descending staircases and for five steps when ascending staircases.

Finally, a note of caution should be added to the definition of the first step when descending (green rectangle in right image in Figure 1). We define this step by its “run,” which is actually part of the floor. We considered this to be the first step because it is where participants have to change their walking behavior to efficiently navigate the staircase. However, this makes the distinction between looking at the first step and looking at the floor somewhat ambiguous. This ambiguity is reported in an earlier study as a reason for disagreement between coders in frame-by-frame analyses (Ioannidou et al., 2017). The gaze position was labeled as being on the first step when it was within the width of a run from the step edge. This approach is not perfect, because the width judgment is somewhat subjective. But luckily ambiguous situations were rarely encountered. The first step was fixated when descending 21 staircases. In 19 of these cases, gaze was very close to the step edge, so there were only two cases in which the width of the run actually had to be estimated. When descending the 13 staircases in which the first step was not fixated, gaze was always directed at a different step and never at an ambiguous position on the floor.

### Data reliability

We computed a task-related estimate of the systematic error and variability of gaze. Red disks with a 3 cm diameter were placed at different positions on each step of an 11-step staircase. We asked three participants who had not taken part in the actual study to look at each target while walking up and down the staircase. We determined the angular distance between the estimated gaze position on the scene and the center of the red disk for each step. We report the average systematic error (right and upward are positive) and average variability (standard deviation across samples for each target) for the *lateral* and *vertical* dimensions separately, together with their standard deviations across both targets and participants (see the Table 1). The reported measures underestimate the reliability because they attribute all deviations from fixating the disk centers to measurement errors.

**Table 1.** Summary of the reliability measures when ascending and descending a staircase. *Lateral* and *Vertical* referred to directions in the video image.

	Ascending		Descending	
	*Lateral*	*Vertical*	*Lateral*	*Vertical*
**Systematic error**	0.23 ± 0.32 degrees	–0.37 ± 1.35 degrees	0.61 ± 0.46 degrees	2.99 ± 2.90 degrees
**Variability**	1.14 ± 0.28 degrees	1.07 ± 0.30 degrees	0.88 ± 0.28 degrees	0.96 ± 0.14 degrees
**Smallest visual extent of step**	11.5 ± 4.3 degrees		5.0 ± 2.5 degrees	

To evaluate how the reliability of the gaze estimates might influence our conclusions, we compared the estimated reliability to the angular sizes of the steps. The mean of the smallest visual angle covered by the center of a fixated step was estimated by averaging the measured extents from the images of eight participants (see the Table 1; the smallest extent was usually mainly in the vertical direction). These measures suggest that our data are quite reliable when ascending staircases but may contain some systematic errors when descending staircases: we may occasionally be attributing gaze to a further step than the participant is actually fixating.

As the three participants’ task for these additional measurements was to look at the red dots, their feet were almost always visible in the video. We could therefore use these measurements to also evaluate the time difference between when the foot first made contact with a step and when the head elevation was minimal (our estimate of stable foot placement). On average, the minimal head elevation was 0.10 seconds after the foot first made contact with the step, both when ascending and when descending. The standard deviation was 0.03 seconds when ascending and 0.07 seconds when descending. The variability between the timing of these two events might be slightly larger in the actual data, because the head orientation is presumably more variable when participants are not instructed to look at red dots on the steps. It is not clear which moment is most relevant when evaluating how many steps ahead the participant is looking, but it is evident that choosing a different moment, such as the moment the foot first made contact with a step, would not make much difference (considering that the average gait cycle during these additional measurements was about 1.1 seconds both when ascending and descending the staircase). 

## Results

### Gaze sequence, fraction of steps that were fixated, and steps looked ahead

**Figure 3.**
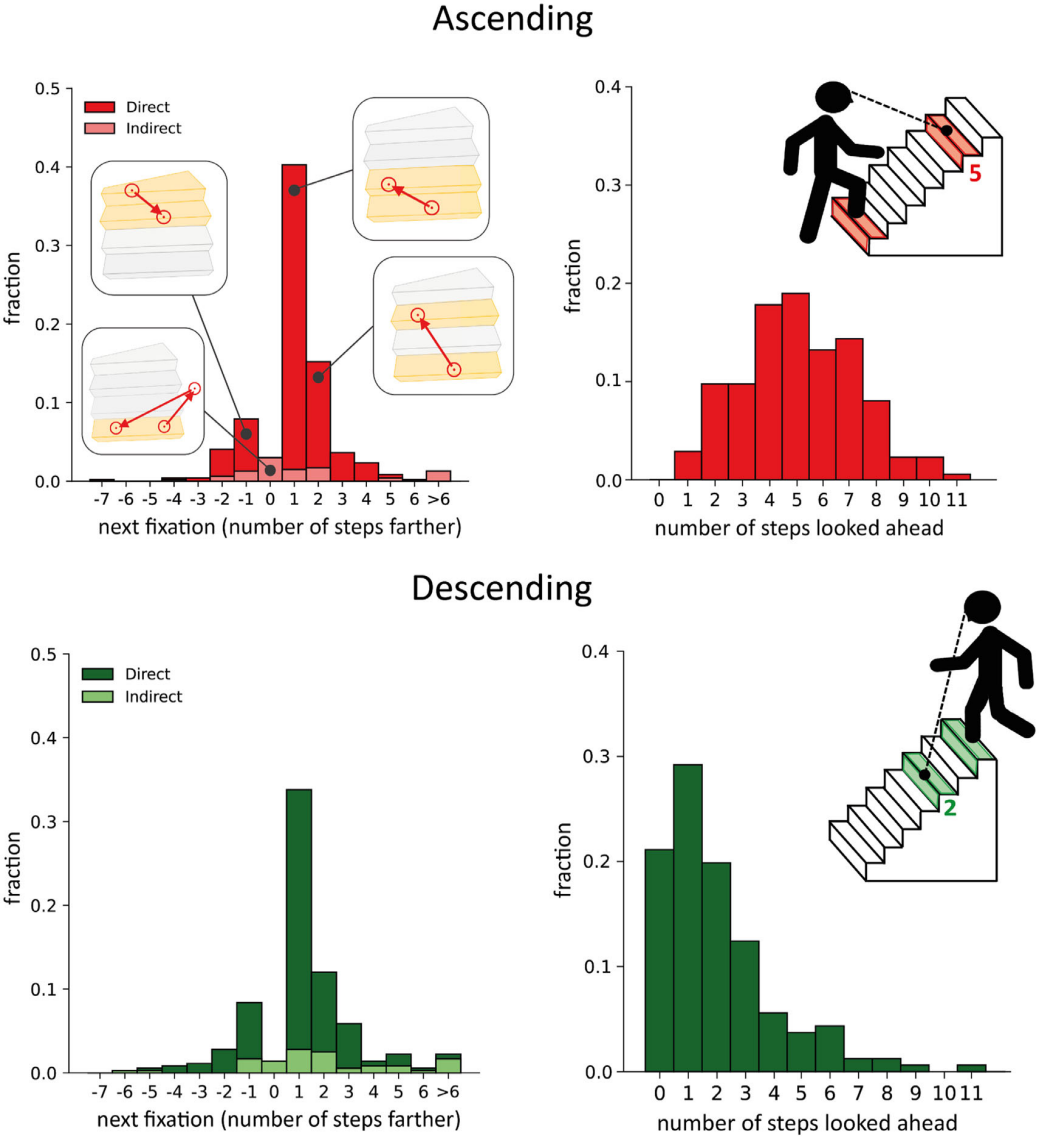
Frequency distribution of how many steps farther participants looked on subsequent fixations when ascending and descending staircases (left panels). *Direct* distributions (dark colors) show how many steps farther the participants looked when subsequent fixations are both on steps. *Indirect* distributions (light colors) show how many steps farther they looked after looking elsewhere. Distribution of the number of steps looked ahead when ascending and descending the staircases (right panels).

**Figure 4.**
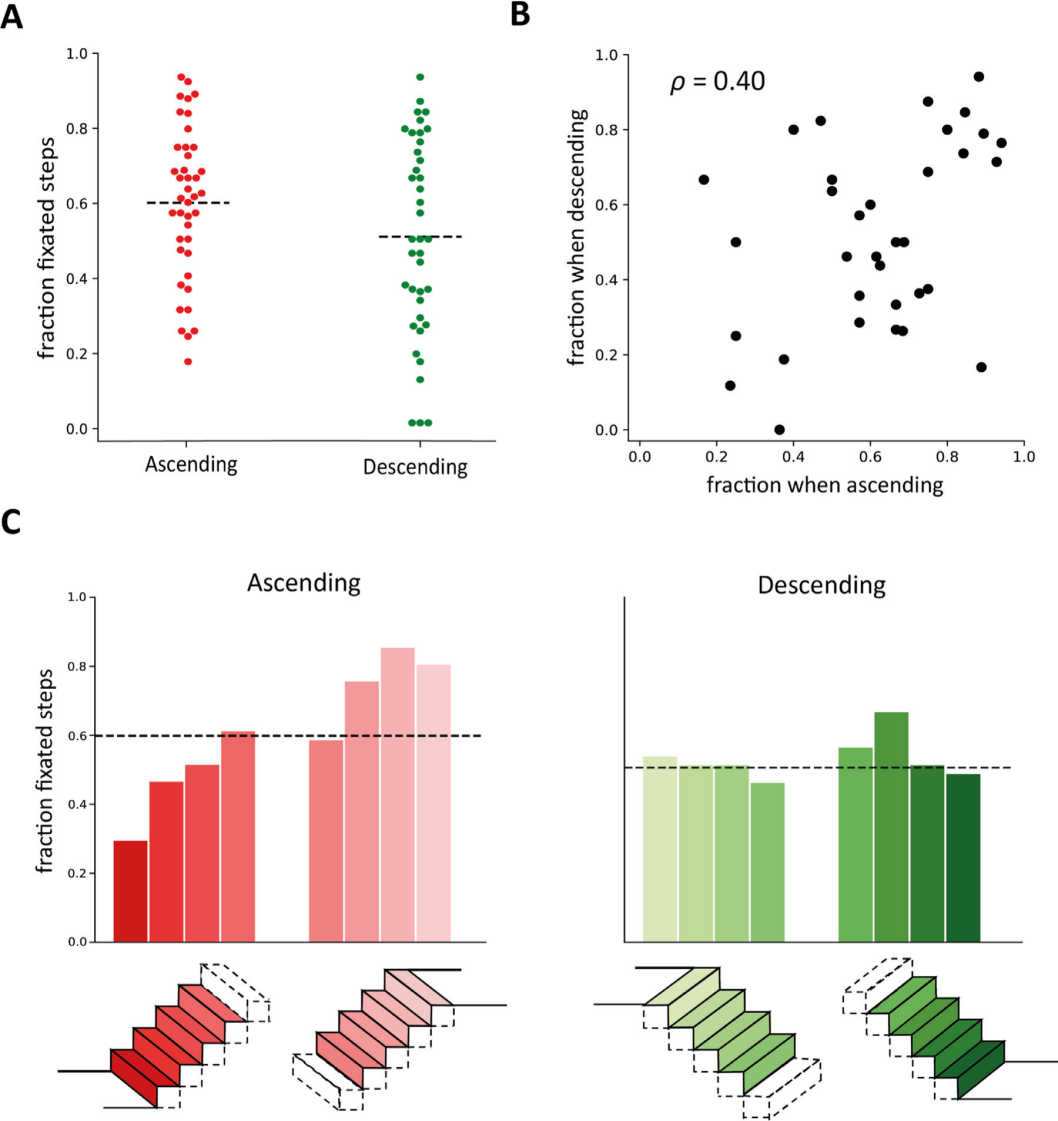
Fixations when ascending and descending staircases. (**A**) Fraction of steps that were fixated. Each dot shows the data for one staircase. The dashed line shows the mean. (**B**) Correlation between the fraction of fixated steps when ascending and descending. Each dot represents an individual participant navigating the same staircase in the two directions. (**C**) Fraction of staircases in which the first four and last four steps were fixated. The black dashed line shows the mean fraction of fixated steps (same values as in **A**).

In general, steps were fixated sequentially: there is a peak at *one* in Figure 3 (left panels). This was so both when ascending and descending the staircases. Sometimes gaze shifted two or more steps ahead or one or more steps back (*Direct*). Occasionally, gaze shifted away from the steps and then back to the same step (value at zero) or some other step (*Indirect*). When ascending staircases, the fixated steps were on average five steps ahead of the step where participants placed their foot stably, with quite a lot of variability in the number of steps. When descending the average was two steps ahead, with a peak at one step ahead (the next step) and quite a few cases in which participants looked at the step that they were stepping onto (see Figure 3, right panels). Not all steps were fixated: despite mainly shifting gaze between successive steps, only about 60% of the steps when ascending and 51% when descending were fixated, with large differences between staircases and participants (Figure 4A). Three participants did not fixate on any step at all when descending, probably relying entirely on peripheral vision to guide foot placement. Interestingly, there was a positive correlation (*p* = 0.40) between the fraction of fixated steps when participants went up and down the same staircase (Figure 4B).

### Gaze distribution across the staircase

In order to explore possible differences in gaze distribution across parts of the staircase, we computed how often the first four steps and the last four steps were fixated, both when ascending and descending the staircase (Figure 4C). Interestingly, contrary to our intuition that it would be beneficial to direct one's gaze at the transition from the ground to the first step, gaze most frequently skipped the first step when ascending the staircase. This was not the case when descending staircases.

### Gaze when approaching the staircase

**Figure 5.**
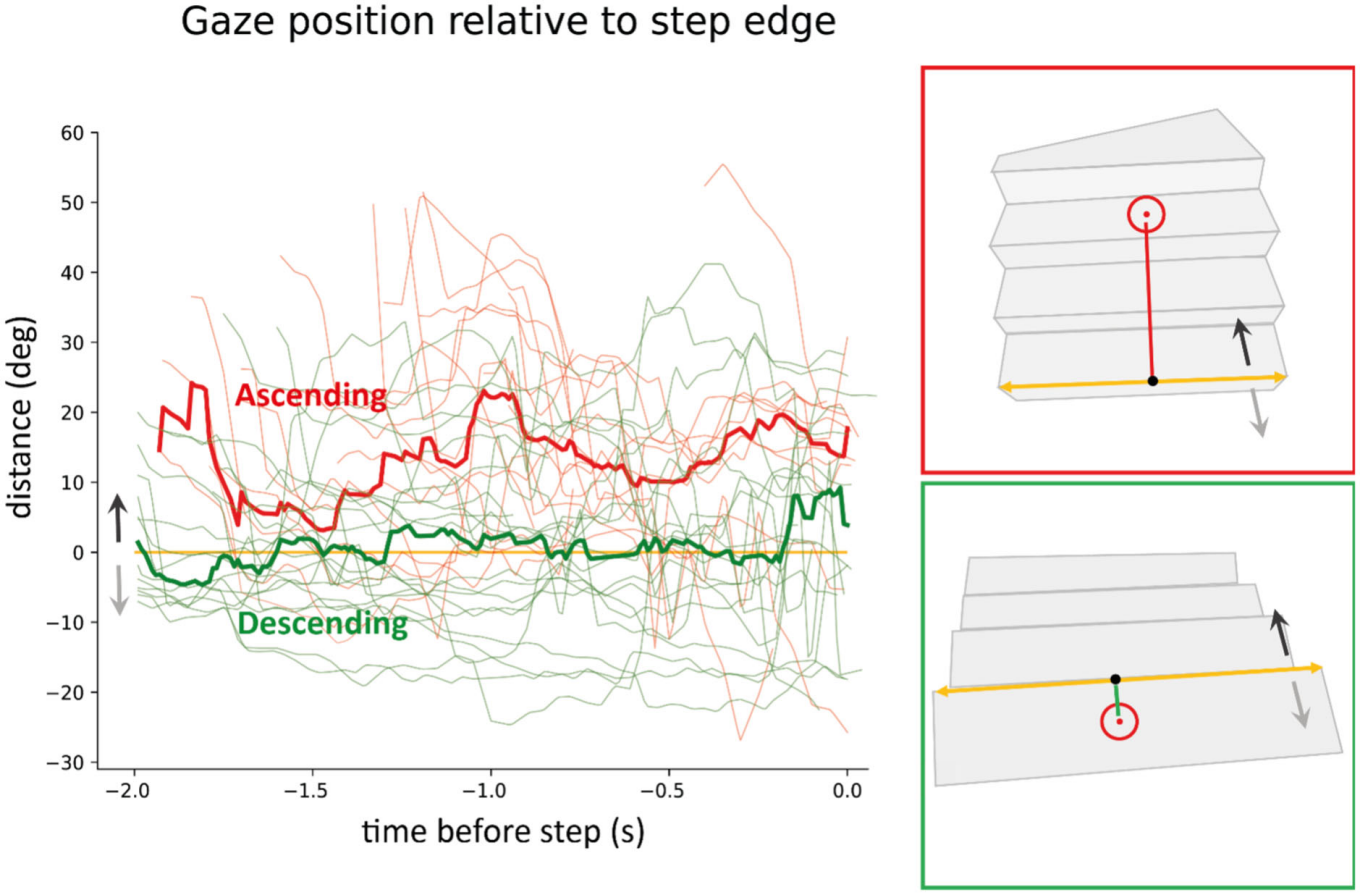
Distance between gaze and the edge of the first step. Positive values represent looking above the edge of the first step (black arrow). Negative values represent looking below the edge (grey arrow). The edge of the first step is shown in yellow. The thin curves show individual staircases. The thick curves show the medians.

We also examined where participants looked as they approached a staircase. The distance was measured between gaze and the closest point on the edge of the first step (see examples in Figure 5). When ascending, the median distance across staircases was positive throughout the period in which participants approached the stairs (see the thick red curve in Figure 5), meaning that gaze was usually directed above the edge of the first step. When descending, the median distance was close to zero (thick green curve), meaning that on average gaze was more or less directed at the edge of the first step when approaching the staircase. There was a lot of variability across participants, both when approaching an ascending and when approaching a descending staircase (see the red and green thin lines in Figure 5), but almost all participants were looking above the first step when approaching an ascending staircase (red thin lines). The tendency to look at the edge of the first step when approaching a staircase going down is consistent with the risk of injury if misjudging the layout at that position. However, the geometry of the two situations might also contribute to the different gaze patterns when approaching staircases going up and down.

To get an impression of the difference in geometry, we computed the mean angular distance between the edge of the first step and the furthest point of the staircase (only considering parts of the staircase that were within the image) as a measure of the visible portion of the staircase during stair approaching. This distance was computed 2 seconds before, 1 second before and when actually stepping stably onto the first physical step when ascending a staircase or the last part of the floor when descending a staircase. It should be noted that the staircases were visible from different perspectives, so this measure does not necessarily represent the vertical extent of the entire staircase when the participant is in front of it, but just the average portion of the staircase visible at these time points. Moreover, whereas the whole staircase was usually visible in the image when descending, only a portion of the staircase was often visible when ascending, especially when getting closer to the first step, so the reported distances when going up underestimate the true distance. The mean distances covered by the staircase for the three above-mentioned time points were 32 degrees, 65 degrees, and 88 degrees, respectively, when going up. The corresponding distances when going down were 12 degrees, 23 degrees, and 35 degrees.

## Discussion

Human behavior may be influenced by the situation and setting in which it is studied (Kingstone, Smilek, & Eastwood, 2008). This is also true for gaze behavior. Some studies have shown that gaze is different when studied under constrained circumstances (in a laboratory setting) as compared to more realistic circumstances (Castelhano et al., 2009; Dicks et al., 2010; Foulsham et al., 2011). In this study, we investigated gaze behavior during stair climbing, a common task in our daily life. So far, gaze behavior during stair climbing has mainly been investigated under highly constrained circumstances with explicit instructions mentioning the staircase. A typical trial in a stair-climbing task starts with participants standing in front of an artificial staircase, with their eyes covered or closed, wearing a safety harness or with a therapist walking next to the participant to aid stability (Miyasike-daSilva, Singer, & McIlroy, 2019; Miyasike-daSilva & McIlroy, 2012; Miyasike-daSilva & McIlroy, 2016; Zietz & Hollands, 2010). Participants are usually instructed to repeatedly walk up and down the same staircase after a go signal from the experimenter (Miyasike-DaSilva et al., 2011; Miyasike-daSilva & McIlroy, 2012; Miyasike-daSilva & McIlroy, 2016; Zietz & Hollands, 2010). This is not how people normally navigate staircases: they usually walk up and down staircases as part of their path to wherever they are going, with many distractions around them and no instructions or precautions emphasizing the staircase.

Laboratory-based studies have shown that when stair climbing is explicitly the task, people tend to fixate around 70% of the steps when going up and 65% when going down (Den Otter et al., 2011) and to spend most of the time looking at stair features along their walking path (Zietz & Hollands, 2010). However, when gaze is directed elsewhere to execute a secondary visual task, participants have no difficulty walking up or down the staircase without directly fixating the steps (Ioannidou et al., 2017; Miyasike-daSilva & McIlroy, 2012; Miyasike-daSilva & McIlroy, 2016), so apparently peripheral vision is sufficient for navigating staircases. The relevance of peripheral vision is supported by studies in which peripheral vision was occluded, showing that people tend to adopt a more cautious behavior when navigating a staircase (Gimunová, Zvonař, Reguli, Ventruba, Ruzbarsky, Duvac, Sagat, & Balint, 2018; Graci et al., 2017; Miyasike-daSilva, Singer, & McIlroy, 2019). This suggests that when not engaged in any other task, people usually direct their gaze at the steps (Miyasike-DaSilva et al., 2011; Zietz & Hollands, 2010) even if this is not strictly necessary for safe navigation (Miyasike-daSilva & McIlroy, 2012; Miyasike-daSilva & McIlroy, 2016). Considering that it is not necessary to direct one's gaze in a particular manner to navigate staircases, we could use this task to test whether the gaze patterns during stair climbing as reported on the basis of studies in constrained circumstances with instructions mentioning the staircase are representative of gaze under more natural circumstances.

Our results showed that participants often looked at each step sequentially, confirming earlier reports from laboratory studies (Den Otter et al., 2011; Miyasike-DaSilva et al., 2011; Zietz & Hollands, 2010). We found that participants also regularly shifted their gaze back to steps that they had already fixated and regularly skipped several steps to fixate further ahead. The sequence of fixations during stair climbing has usually been described in terms of sequentially looking two to four steps ahead when going up staircases and sequentially looking within four steps ahead when going down (Miyasike-DaSilva et al., 2011; Zietz & Hollands, 2010). In more natural circumstances, looking more than four steps ahead has been reported when ascending (Ioannidou et al., 2017) or descending (den Otter et al., 2011) staircases. Moreover, shifting gaze to a step that is several steps ahead of the one that is currently fixated, and shifting gaze to steps that are closer than the one that is currently fixated, has also been reported or can be inferred from the results of previous studies (Den Otter et al., 2011; Miyasike-DaSilva et al., 2011). Thus, overall, our findings (looking at each step sequentially most of the time and occasionally looking back at previous steps or skipping several steps to look further ahead) are quite consistent with previous studies that investigated stair walking under much more constrained circumstances.

We found that people often looked four or five steps ahead when ascending staircases, which is slightly more than the number reported in some laboratory studies (Miyasike-DaSilva et al., 2011), but in line with a previous study conducted under more natural circumstances (Ioannidou et al., 2017). Interestingly, we found that people looked much closer when descending staircases, with a peak at one step ahead and an average of two steps ahead. This is different from the four steps ahead previously reported when descending staircases (Den Otter et al., 2011; Miyasike-DaSilva et al., 2011; Zietz & Hollands, 2010). It is unlikely to result from a systematic error in measuring gaze, because systematic errors are likely to make us overestimate the number of steps ahead rather than underestimating it (see the Table 1). Another possibility is that we misjudged the step on which participants were standing, because we rely on data from the *IMU* in the eye tracker to estimate when the head position reaches a minimum, which gives a value that is slightly after the foot first makes contact with the step. It may be further delayed because the head rotates and the *IMU* is in the eye tracker, which is on the head. However, because participants often rotated their head downward (so that their foot was visible in the image) when descending staircases, we could sometimes check in the image whether they were really fixating the step onto which they were placing their foot (at the moment defined by the *IMU* data), and this was usually clearly the case. It is therefore more likely that the smaller number of steps looked ahead in this study is primarily due to the Dutch staircase structure. The staircase steepness in previous studies was between 30 degrees and 34 degrees (Den Otter et al., 2011; Miyasike-DaSilva et al., 2011; Zietz & Hollands, 2010). Traditional Dutch staircases have a steepness of about 45 degrees. The difference in the number of steps that participants looked ahead when ascending and descending staircases may therefore be a result of the steeper staircases used in the current study, rather than being the result of the more natural circumstances. The comparison between the actual number of steps looked ahead in this study and in earlier studies should be taken with caution, as some studies do not explicitly mention the procedure they use to compute this measure (Ioannidou et al., 2017; Miyasike-DaSilva et al., 2011) and others use a different procedure (Zietz & Holland, 2010; see Gaze sequence, fraction of steps that were fixated and steps looked ahead).

Although fixating one step after the other was the most common gaze pattern, participants only directed their gaze at about half the steps (about 60% and 51% when going up and down, respectively). Higher percentages have previously been reported (about 70% and 67% when going up and down, respectively, in Den Otter et al., 2011), suggesting that participants may look at fewer steps when the settings are more familiar. We found a high variability in the fraction of fixated steps across participants and staircases. The positive correlation between the fractions of fixated steps when going up and down (see Figure 4B) suggests that the high variability is not (only) caused by coincidental distractions. Some people may generally rely more on central vision while others readily rely on peripheral vision when navigating staircases. However, as almost each participant navigated a different staircase, the difference may be due to the staircase structure rather than the participant, with some kinds of staircase requiring more central vision than others. We found no obvious relationship between the fraction of fixated steps and the steepness of the staircase (as judged visually from the scene videos), the presence of objects on the steps or on the handrail, the presence of open rises (as in Figure 2A) or of having walls on both sides of the staircase (as in Figure 2B). We interpret the finding that participants did not look at many of the steps before stepping on them as suggesting that peripheral vision is used to guide foot placement on such steps. But participants might also know where to place their foot based on their extensive experience with that staircase. They might know where the step is with respect to other fixated structures in the room, or with respect to the current position of the foot that they are standing on. Partly relying on experience would explain why fewer steps were fixated than in previous studies, where participants were less familiar with the staircase.

Visual guidance may be particularly important during transitions from the ground to the first step, as this is where people need to adjust their locomotion (Graci et al., 2017; Miyasike-daSilva & McIlroy, 2016; Telonio et al., 2014). However, transition steps are not fixated more than middle steps in a controlled setting (Miyasike-DaSilva et al., 2011). We tested whether this is also true in a more familiar and uncontrolled scenario. Contrary to the idea that it might be particularly beneficial to direct one's gaze at this portion, the first step was fixated even less frequently than other steps when ascending staircases. It was fixated as all others when descending staircases. The difference is already evident when approaching the staircase: when approaching the first step to descend a staircase, participants looked extensively at the beginning of the staircase, close to the first step's edge (see Figure 5). When approaching the first step to ascend a staircase they generally looked above the edge of the first step. As already mentioned, this difference is probably related to differences in the geometry of the view of the staircase, with the staircase filling a much smaller part of the visual field while approaching it to go down. This probably makes it more difficult to deduce the position of the first step from visual information from later steps. However, the cost of making an error is also larger when descending.

In sum, in line with previous studies, people tend to look at the steps sequentially. Thus, this does not require controlled circumstances or specific instructions. We quantify the flexibility whereby gaze skips several steps, returns to previously fixated steps, or is even never directed at steps. The occurrence of such gaze behavior can be inferred from some previous studies (Miyasike-DaSilva et al., 2011), but gaze has mainly been discussed in terms of the predominant sequential looking ahead pattern, without providing details about the non-sequential shifts of fixation. We found an overall lower fraction of fixated steps than earlier reports, which may suggest that people tend to look less at the staircase in more familiar circumstances. We also found a high variability in the fraction of fixated steps, possibly related to different visual strategies across individuals or kinds of staircases.

### How reliable are our findings?

As in all studies using a manual frame-by-frame analysis, the definition of our area of interest (a step) and the way ambiguous situations are handled is to some extent arbitrary. We tried to be as explicit as possible about our definitions and how we dealt with ambiguous situations. As in all eye tracking studies, the resolution of the eye tracker can limit the reliability. We report measures of the systematic error and variability of gaze when ascending and descending staircases based on separate measurements in which participants were instructed to look at particular landmarks. We did so under circumstances that closely resemble those that we are interested in, because the resolution is likely to depend on the circumstances. The results of these measurements suggest that the resolution during stair ascending is good enough for our analysis, but the resolution when descending needs to be considered when interpreting the data. This is not only the result of a lower estimated resolution of the eye tracker when descending, but also because of the smaller angular extent of a step when descending. The eye tracker may be less reliable when descending because gaze is directed more eccentrically with respect to the head, despite the head being tilted further downward (Miyasike-daSilva et al., 2019). Evidence of this can be seen in how often steps needed to be guessed because gaze was outside the scene camera when going down (see Ambiguous situations).

On the bright side, it is also likely that we underestimated the reliability of the eye tracker because our measure is based on the assumption that participants adhere to the instruction to fixate the centers of the landmarks. Consequently, any deviation in gaze (while walking down the staircase) is attributed to measurement error. We measured gaze while walking rather than having participants stand still to fixate the landmarks because we wanted to measure resolution under similar conditions to the actual experiment.

**Figure 6.**
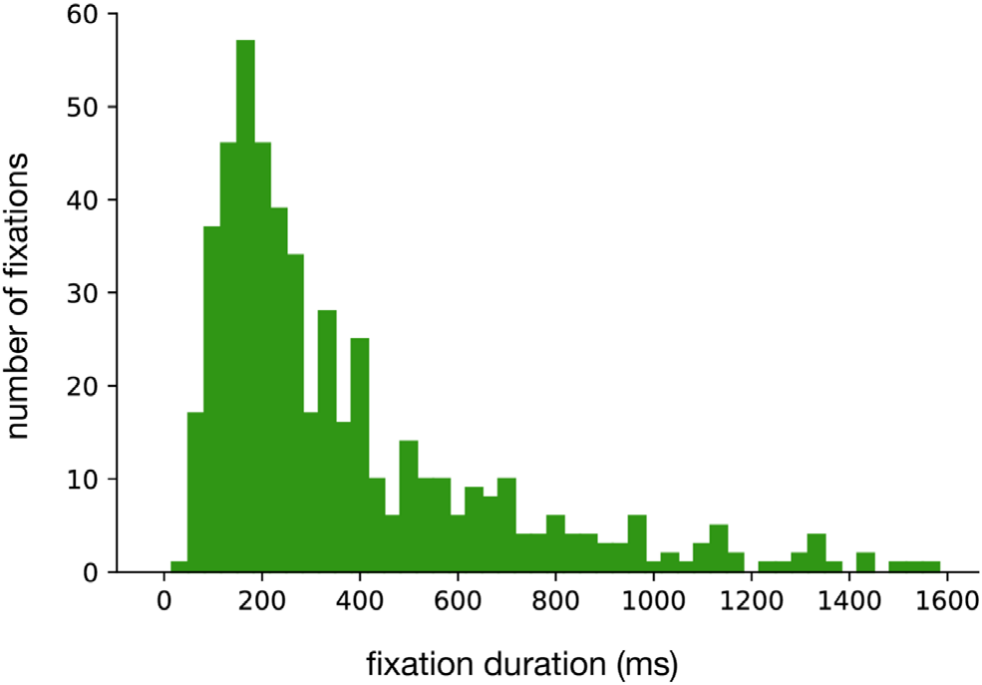
Distribution of fixation durations in the time window between the first fixation on any step and the last fixation on the last fixated step (irrespective of where participants were fixating).

We considered gaze that was directed at about the same place on two successive frames to be a fixation. This is a minimum, because, if gaze is only directed somewhere on a single frame, it might actually be midway during a saccade. Normally, fixations last more than two frames (66 ms), so relying on this minimal duration might include some instances that are not really fixations. On the other hand, requiring three frames would mean that fixations of just less than 100 ms could be missed, which might reduce our estimate of the number of fixated or sequentially fixated steps. To examine whether this is an issue worth worrying about we plot a histogram of the frequency of fixation durations as determined using an automatic fixation classification (available at the location indicated in the Data Availability Statement). It is evident from this plot (Figure 6) that requiring three frames would make little difference. Note that excluding short fixations can further decrease the number of fixated steps in comparison with earlier studies, but it cannot increase it.

Finally, for the measures for which we compute a distance in degrees from the scene videos (the distance from the first step edge while approaching the staircase, and the step and staircase visual extents), the images were not corrected for distortions introduced by the optics of the Pupil Invisible scene camera. This means that the values we reported are overestimated in the central part of the scene and underestimated in more eccentric parts. Because of this, the difference between the visual extent of the staircase when going up and down may be even larger than that reported in Gaze when approaching the staircase.

## Conclusion

Despite the limitations that accompany eye movement measurements in unconstrained settings (Hessels, Niehorster, H, 2020), we were able to study gaze during stair climbing in a setting that is very close to that encountered in daily life. Participants were obviously aware that their gaze was being recorded, but by avoiding mentioning staircases in the instructions and conducting the research on a variety of staircases in different familiar environments, we probably obtained a description of gaze behavior that is more representative of that encountered in daily life. Many aspects of gaze were similar to those reported in earlier studies under highly constrained circumstances, which validates the use of such circumstances, but there were also some differences that are worth further exploring.
